# CASE SCENARIOS OF CANADIAN MEDIC-ALERT SUBSCRIBERS WITH DEMENTIA WHO GO MISSING

**DOI:** 10.1093/geroni/igad104.0166

**Published:** 2023-12-21

**Authors:** Christine Daum, Vanessa Vahedi, Emily Rutledge, Adebusola Adekoya, Antonio Miguel-Cruz, Lili Liu

**Affiliations:** University of Waterloo, Waterloo, Ontario, Canada; University of Waterloo, Waterloo, Ontario, Canada; University of Waterloo, Waterloo, Ontario, Canada; University of Waterloo, Waterloo, Ontario, Canada; University of Alberta, Edmonton, Alberta, Canada; University of Waterloo, Waterloo, Ontario, Canada

## Abstract

With the increasing prevalence of dementia worldwide, it is expected that incidences of getting lost and going missing among persons living with dementia due to wayfinding challenges will also increase. Despite this, there is currently a gap in accessible education materials specific to risk factors and incidents of getting lost and going missing, aimed at first responders and family care partners. Case scenarios can effectively convey the circumstances of recurring events while also accounting for lived experiences. The purpose of this project was to develop case scenarios informed by real-life missing incidents that involve persons living with dementia in Canada. Qualitative description and conventional content analysis were used to analyze summary notes of missing incidents (n=515) obtained from Medic-Alert Foundation Canada hotline database. Summary notes contained: demographics of the missing person, their living situation and circle of support, events leading up to incident, by whom and where they were found, their health condition, and how they were re-united with their care partners. Case scenarios were developed that reflected the common (e.g., going missing while on foot, being found on the street) as well as less common (e.g., going missing while driving, being found seriously injured). Five experts (person living with dementia, care partner, health care professional, support providers) reviewed these scenarios to ensure relevance. Case scenarios can raise awareness of the circumstances encountered during missing incidents. The scenarios can be used by first responders and care partners to learn how to respond to missing incidents and receive support.

